# Expression of Concern: ANRIL promotes chemoresistance via disturbing expression of ABCC1 by regulating the expression of Let-7a in colorectal cancer

**DOI:** 10.1042/BSR20180620_EOC

**Published:** 2025-09-30

**Authors:** Zhen Zhang, Lifeng Feng, Pengfei Liu, Wei Duan

**Affiliations:** 1 The Department of Gerontology, Yeda Hospital of Yantai City, Yantai, China; 2 The Department of General Surgery, Shangluo Central Hospital, Shangluo, China; 3 The Department of Gastroenterology, Yan’an University Affiliated Hospital, Yan’an, China; 4 The Department of Oncology, Yan’an University Affiliated Hospital, Yan’an, China

*Bioscience Reports* has been made aware of potential issues surrounding the scientific validity of this paper and is hence issuing an Expression of Concern to notify readers whilst the Editorial Office investigates. Unexpected similarities have been noted between the Figure 2G bottom si-NC colony assay, rotated 90 degrees, and the Figure 5C OSCC3/si-NC colony assay from Zhang et al. 2017 (doi: 10.1038/s41598-017-13431-y). The authors have been contacted for comment, and we have not yet received a response or the requested raw data.

